# Review of ^18^F-FDG Synthesis and Quality Control

**DOI:** 10.2349/biij.2.4.e57

**Published:** 2006-10-01

**Authors:** S Yu

## Abstract

This review article covers a concise account on fludeoxyglucose (^18^F–FDG) synthesis and quality control procedures with emphasis on practical synthesis Currently, ^18^F–FDG is the most successful PET radiopharmaceutical so far. The advancement in synthesis and quality control of ^18^F–FDG, together with its approval by the US FDA and the availability of reimbursement, are probably the main reasons for the flourish of clinical PET over the last 20 years. ^18^F–FDG can be synthesised by either electrophilic fluorination or nucleophilic fluorination reaction. Nucleophilic fluorination using mannose triflate as precursor and Kryptofix or tetrabutylammonium salts (TBA) is widely used because of higher yield and shorter reaction time. The quality control requirements of ^18^F–FDG can be found in United States Pharmacopeia (USP), British Pharmacopeia (BP), European Pharmacopeia (EP) and the Chemistry, Manufacturing, and Controls (CMC) section from United States Food and Drug Administration (US FDA) PET draft guidance documents. Basic requirements include radionuclidic identity, radiochemical purity, chemical purity, pH, residual solvent, sterility, and bacterial endotoxin level. Some of these tests (sterility, endotoxins and radionuclidic purity) can be finished after the ^18^F–FDG has been released. Although USP, BP and EP do not require filter membrane integrity test, many laboratories perform this test as an indirect evident of the product sterility. It is also interesting to note that there are major differences in ^18^F–FDG quality requirements among USP, BP, and CMC.

## INTRODUCTION

^18^F–FDG is a glucose analogue in which the hydroxyl group on the 2–carbon of a glucose molecule is replaced by a fluoride atom. Like glucose, ^18^F–FDG is taken up into living cells by facilitated transport and then phosphorylated by hexokinase. Unlike glucose, ^18^F–FDG cannot undergo further metabolism because the hydroxyl group at the 2–carbon is a requirement for the process [1–2]. Nevertheless, ^18^F–FDG is a good indicator of glucose uptake and cell viability.

The uptake of glucose analogues into living cells also depends on modifications of various carbons at different positions. It has been shown that the specificity of 3–deoxyglucose (3–DG) and 4 deoxyglucose (4–DG) towards hexokinase reduced by 100–fold [3], hence 3–DG and 4–DG were not retained inside the cells. Similary, 3–fluoro–deoxyglucose and 4–fluoro-deoxyglucose do not accumulate in living cells as much as ^18^F–FDG. Although the nucleophilic substitution reaction is more widely used nowadays, the electrophilic fluorination reaction has an important place in the synthesis of ^18^F–FDG.

## SYNTHESIS OF ^18^F-FDG BY ELECTROPHILIC FLUORINATION

**Figure 1:**
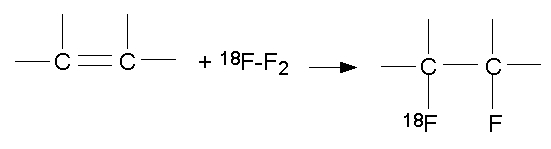
Electrophilic fluorination.

**Figure 2:**
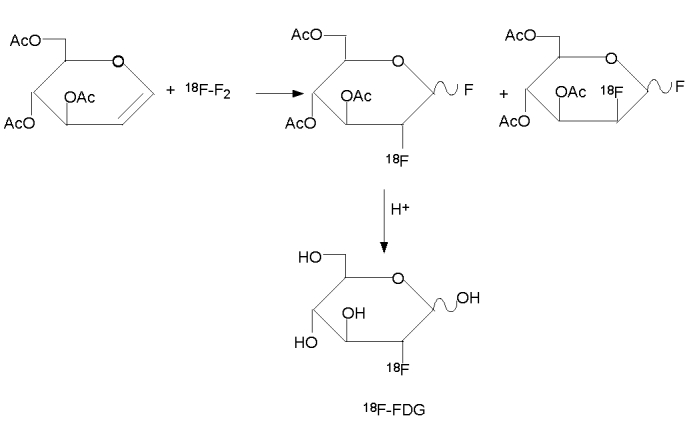
Synthesis of 18F-FDG by electrophilic fluorination.

The first synthesis of ^18^F–FDG was carried out in Brookhaven National Laboratory by Wolf et al in 1976 by electrophilic fluroination [4]. As shown in Figure 1, electrophilic fluorination refers to the addition of fluorine atoms across a double bond, producing a difluoro derivative of the parent compound. The electrophilic fluorination by Wolf et al involved the use of 3, 4,6–tri–O–acetyl–D–glucal as precursor. The glucal was treated with ^18^F–F_2_ to produce a 3:1 mixture of ^18^F labeled difluoro–glucose and difluoro–mannose derivatives. The difluoro–glucose derivative was separated and hydrolysed with hydrochloric acid to form 2–fluoro–2–deoxyglucose (Figure 2). The yield was 8% and the synthesis time was 2 hours [4].

Despite the low yield and long synthesis time, the Brookhaven team was able to collaborate with The Hospital of the University of Pennsylvania to map glucose metabolism in human brain [4]. This was the first ^18^F–FDG trial in human.

Several improvements to the electrophilic fluorination described above were made thereafter. One of the most useful modifications was the use of acetylhypofluorite ^18^F–CH_3_CO_2_F. The acetylhypofluorite can be produced *in situ* from ^18^F–F_2_. The yield was higher and the synthesis reaction was easier to control [4–6 ].

The major limitation of electrophilc fluorination was that only 50% of the radioactive fluorine atoms were incorporated into the precursors. In addition, the ^18^F–F_2_ was produced from a Neon gas target with 0.1% to 1% of fluorine gas via a ^20^Ne(d,α)^18^F reaction. The specific activity is lower due to the presence of the non–radioactive fluorine gas. The maintenance and operation of a Neon target is troublesome and the yield of ^18^F^–^ was much lower than with the ^20^Ne(d,α)^18^F reaction than with the ^18^O(p,n)^18^F^–^ reaction. [4, 7–8]

## SYNTHESIS OF ^18^F-FDG BY NUCLEOPHILIC FLUORINATION

Many attempts have been made to develop a nucleophilic substitution for the synthesis of ^18^F–FDG. This included the use of ^18^F–CsF, ^18^F–Et_4_NF, and ^18^F–KHF [4, 9–14]. But the major breakthrough was reported in 1986 by Hamacher et al who had used Kryptofix 222^TM^ as a catalyst [15]. The reaction had a consisten yield of over 50% and the reaction time was shortened to 50 min.

**Figure 3:**
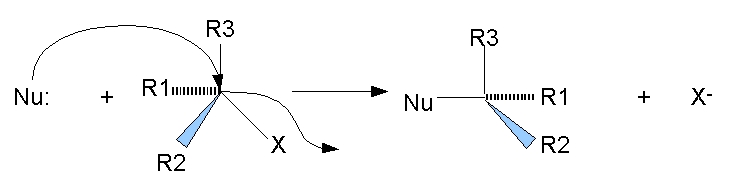
Nucleophilic substitution: Nu = nucleophilic molecule, X = leaving group.

Nucleophilic substitution is a chemical reaction involving the addition of a nucleophilic molecule (highly negatively charged molecule) into a molecule with a leaving group (electron drawing group attached to the parent molecule through an unstable chemical bond). Figure 3 is a general scheme for an SN2 nucleophilic substitution reaction. The nucleophilic molecule has a high affinity towards the relatively electron deficient center in the parent molecule created by the electron pulling leaving group. As a result, the nucleophilic molecule forms a covalent bond with the parent molecule and displaces the leaving group. The stereo–configuration of the parent molecule is also changed.

**Figure 4:**
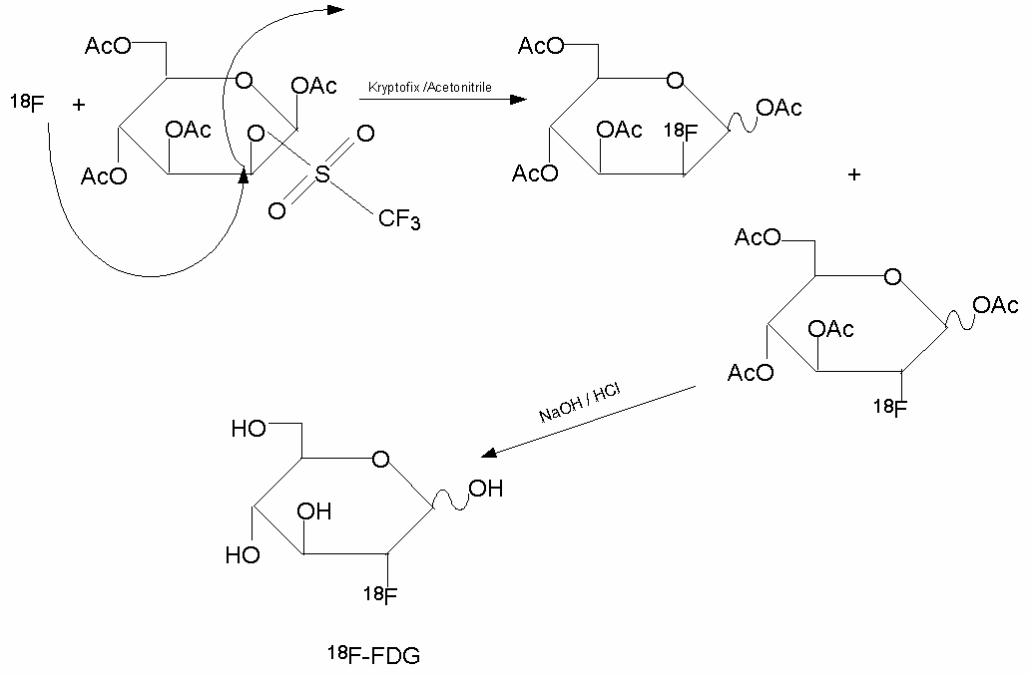
Synthesis of ^18^F-FDG by nucleophilic substitution.

In the synthesis of ^18^F–FDG, ^18^F ion is the nucleophile. The precursor is mannose triflate in which the 1,3,4,6 position carbons of a mannose moleucle are protected with an acetyl group and triflate is the leaving group at the 2–carbon. In the presence of Kryptofix 222^TM^ as catalyst and acetonitrile as solvent, ^18^F ion approaches the mannose triflate at the 2–carbon, while the triflate group leaves the protected mannose molecule to form ^18^F–FDG (Figure 4).

Although synthesis of ^18^F–FDG can be carried out in different computer controlled automatic synthesizers, the nucleophilic process proceeds in roughly same stages:

### Removal of ^18^F from the ^18^O^-^ water coming out from the cyclotron target.

**Figure 5:**
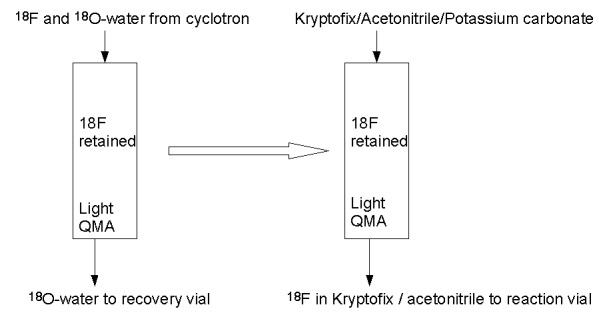
(A) Retention of 18F-FDG in light QMA ion exchange column (B) elution of 18F from light QMA ion exchange column.

Fluorine has a high hydration energy, so water is not a suitable solvent in this synthesis. Polar aprotic solvent such as acetonitile should be used in an SN2 nucleophilic substitution reaction. Since ^18^F^–^ is produced by a ^18^O(p,n)^18^F^–^ reaction, it is necessary to isolate the ^18^F ion from its aqueous environment. The most convenient way to isolate is to use a light QMA (Quaternary ammonium anion exchange) Sep–Pak column (Accell Plus QMA Sep–Pak^TM^). The ^18^F^–^ is retained by or via an ion–exchange reaction and allowed the ^18^O–water to flow through. The retained ^18^F^–^ is then eluted with an acetonitrile solution of Kryptofix and potassium carbonate (Figure 5).

In an aqueous environment, any negatively charged ions must be accompanied by positively charged counterparts. Usually, the ^18^F^–^ washed out from the cyclotron target is accompanied by traces of metal ions from the surface of the target body. When passing through the light QMA anion exchange ion, the ^18^F ^–^ is retained and the metal ions will be lost in the ^18^O^–^ water. Hence, it is necessary to introduce a positively charged counter ion to restore the ^18^F^–^ reactivity before evaporation of residual ^18^O^–^ enriched water [16].

**Figure 6:**
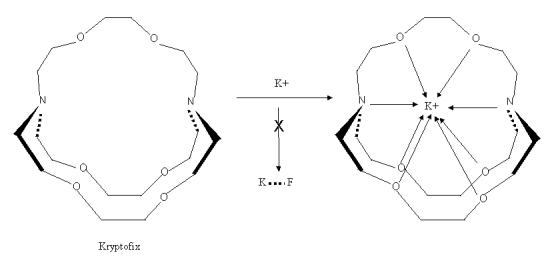
Kryptofix 222 ™ and K^+^.

Several types of positively charged counter ions have been used, including large metal ions such as rubidium or cesium; potassium ion complexed by a large ring structure such as Kryptofix 222^TM^ and tetrabutylammonium salts [16–17]. Kryptofix 222^TM^ is a cyclic crown ether (Figure 6), which binds the potassium ion, preventing the formation of ^18^F–KF. Thus, potassium acts as the counter ion of ^18^F^–^ to enhance its reactivity but does not interfere with the synthesis.

Since Kryptofix 222^TM^ causes apnea and convulsion, all automatic synthesis modules have multiple removal steps so that there is only negligible amount of Kryptofix in the final ^18^F–FDG products. Tetrabutylammonium salts (TBA) are also widely used as catalyst in place of Kryptofix 222^TM^ [18].

Logically, the addition of a counter cation also includes the addition of another anion. The carbonate anion is most widely used because it is less likely to interfere with the synthesis [16].

### Evaporation of residual ^18^O^-^ water from the ^18^F with acetonitrile

After the ^18^F^–^ is eluted into reaction vessel, it is necessary to evaporate any residual water from the solution. The advantage of using acetonitile as the eluting solvent is that it forms an azeotropic mixture with water. Evaporation of the acetonitrile in a nitrogen atmosphere will at the same time remove any residual ^18^O^–^ water escaped into the reaction vessel together with the ^18^F. Most of the ^18^F–FDG automatic synthesizers perform the acetonitrile evaporation step several times to ensure all the residual ^18^O^–^ water is removed. All components of the synthesis system are also rinsed with acetonitrile to remove moisture. Dry nitrogen (moisture content less than 3 ppm) should be used in the synthesis

### Addition of mannose triflate into the ^18^F^-^ with acetonitrile.

**Figure 7:**
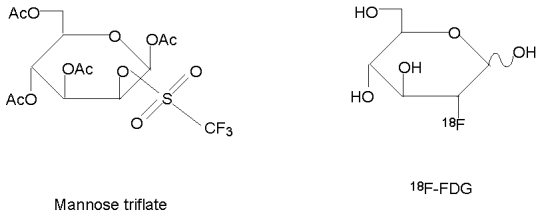
Structures of Mannose triflate and ^18^F-FDG.

The nucleophilic substitution takes place in this stage. After the evaporation of any residual water, the precursor is added to the ^18^F^–^. The choice of precursor depends on the ease of preparation, ease of producing the final product, consistency, yields, and so on. The most commonly used precursor molecule in synthesis of ^18^F–FDG is 1,3,4,6–O–Acetyl–2–O–trifluoro-methanesulfonyl–beta–D–mannopyranose (mannose triflate). Its structure (Figure 7) is similar to that of FDG, except with a triflate group at the 2 carbon position and acetyl groups at 1,3,4,6 position carbons via ester bonds, which can be readily broken at a higher or lower pH. The use of acetyl groups is to protect the hydroxy groups so that fluorination would not occur at these positions. The ^18^F ion approaches the mannose triflate at the 2 position carbon, while the triflate group leaves the protected mannose molecule to form ^18^F–FDG (Figure 4). After the nucleophilic replacement of the triflate group by ^18^F^–^, the acetyl groups can be easily removed by hydrolysis to give rise to ^18^F–FDG

The choice of leaving a group is an important consideration. A good leaving group should have the properties of leaving the parent molecule readily. Once it departs from the parent molecule, its negative charge is stabilised by delocalisation and it will not re–enter the parent molecule.

Commonly available leaving groups includes triflates, tosylates, mesylates among others. The choice of leaving a group depends on the nature of the reaction, the solvent, the stability of the precursor, and so on. All of the leaving groups listed in Table 1 except chlorides have been used in radio–fluorination reactions. In the synthesis of ^18^F–FDG, trifaltes produces a higher and more consistent yield at about 50 to 60% [16].

**Table 1:** A comparison of various leaving groups

**Leaving group Properties**	
Trifates	very good leaving group
Toyslate	
Mesylate	
Iodide	moderate leaving group
Bromide	
Chloride	poor leaving group

### Hydrolysis to remove the protective acetyl groups to form ^18^F-FDG

The final step of the synthesis is to remove the protective acetyl groups on the 1,3,4,6 position carbons. This can be accomplished by either using hydrochloric acid (acid hydrolysis) or sodium hydroxide (base hydrolysis). Acid hydrolysis requires a longer time and higher temperature. Base hydrolysis, which is more commonly used currently, is faster and takes place at room temperature. One of the improved base hydrolysis is to adsorb the 1,3,4,6 acetyl protected ^18^F labeled 2 dexoyglucose on to a C–18 reverse phase column. All other impurities can be removed by rinsing heavily with water. Sodium hydroxide is added to the column so that the base hydrolysis occurs on the column surface. The final ^18^F–FDG product can be eluted with water while the unhydrolysed or partially hydrolysed 1,3,4,6 acetyl protected ^18^F labeled 2 dexoyglucose remains on the column [19].

### Purification of the final ^18^F-FDG product.

Purification of the final ^18^F–FDG can be performed with a series of anion exchange column, C–18 reverse phase column and alumina column. Most automatic synthesizers can produce ^18^F–FDG of over 95% routinely.

## QUALITY CONTROL OF ^18^F-FDG

The quality requirements of ^18^F–FDG are set out in various pharmacopeia including the USP [20], BP [21], EP [22], etc. The US FDA has also published a draft Chemistry, Manufacturing and Controls (CMC) document concerning ^18^F–FDG [23]. It should be noted that the quality control requirements of ^18^F–FDG differ among these references. An excellent comparison between them can be found elsewhere [24]. In Asia, Taiwan has established an official guidelines for the compounding of PET drug products, as well as for the quality control of ^18^F–FDG.

Different countries my adopt a different set of standards. The BP is described in this article solely because this is the standard adopted by the author’s country. Table 2 lists the quality control tests required by BP [21]. Due to short half–life of ^18^F–FDG, not all the listed tests can be completed before release of the ^18^F–FDG product. The BP allows the ^18^F–FDG to be released before the radionuclidic purity test, bacterial endotoxin test, and sterility test are finished.

**Table 2:** quality control tests of ^18^F-FDG listed in BP

**Test**	**Method**	**Acceptance Criteria**
Character	Not specified	Clear, colourless or slightly yellow liquid
Identification	Gama- Gamma-spectrum	Photon energy of 0.511Mev or 1.022MeV
	Half-life measurement	105 to 115 min
	Examine the chromatogram peak from radiochemical purity test	The product should have the same retention time as the reference solution
pH	Not specified	4.5 to 8.5
Chemical Purity:		
2-FDG	HPLC	The area of the 2-FDG peak is not greater than the area of the reference peak (10mg/maximum injected dose in mL)
Kryptofix	Colourimetirc	The test spot should not be darker than the reference spot
tetra-alky ammonium salt	HPLC	The area of the product peak is not greater that the area of the reference peak (2.75 mg / maximum injected dose in mL)
Residual solvent:		
Acetonitrile	Not specified	Less than 4.1mg per maximum dose volume injected
Radionuclidic purity	Gama-spectrum	Photon energy of 0.511Mev or 1.022MeV
	measurement of half-life	105 to 115 min
Radiochemical Purity	HPLC TLC	Not less than 95% of the total radioactivity
Sterility	Standard sterility test according to BP	No bacterial growth
Bacterial Endotoxin	Not specified	175 international unit/maximum dose in mL
Radioactivity	Measurement in calibrated dose calibrator	----

There are other tests not listed in the BP, but may be of significance. The BP does not list a test for ethanol, which is widely used in the synthesis of ^18^F–FDG. Both USP and BP do not list the membrane filter integrity test. However, the test is essential as an indirect evidence of the ^18^F–FDG product sterility because the sterility test result will not be available until much later.

### Character:

Although the BP does not specify the test method, it is obvious that a visual inspection of the ^18^F–FDG is implied. The product should be observed behind adequate shielding. While BP allows a sightly yellow colour, this may indicate the presence of impurities. An ^18^F–FDG product should only be clear and colourless.

### Identity (radionuclidic and radiochemical)

In BP, the tests for radionuclidic identity and radiochemical identity are the same tests for radionuclidic purity and radiochemical purity. The radionuclidic identity can be confirmed either by obtaining a gamma spectrum or measuring the half life of the product. However, the photon energy of 0.511 MeV and the sum peak at 1.022 MeV are common features to positron emitters. Hence, obtaining a gamma spectrum may not be adequate in confirming the presence of ^18^F^–^ [24].

Measurement of the half–life can be carried out by measuring the same test solution in the same dose calibrator at 2 or more time points. The half–life is then calculated by plugging the results into the radioactivity decay equation. The BP does not specify the time interval between each measurement, but it should be long enough to allow a significant decay. The author suggests a minimum decay period of of 20 to 30 min. Other experts have established that a minimum of 10 min is necessary [24]. The measurement of half–life is a more reliable method in confirming the presence of ^18^F^–^.

In BP, the radiochemical identity can be confirmed either by HPLC or TLC. The TLC is easier, but as accurate and reliable as the HPLC. However, TLC may take longer time. For the test of ^18^F–FDG, the TLC stationary phase is TLC–SG and the mobile phase is acetonitrile : water (95%:5% v/v). The Rf of the ^18^F–FDG, free ^18^F^–^, and acetylated ^18^F–FDG are abut 0.45, 0.0 and 0.8 to 0.95 respectively. It should be noted that TLC results can vary according to different brands of TLC plates and operation conditions. It is therefore important to use the same brand of TLC–SG plate and freshly prepared mobile phase if possible. When plates with a new batch number (from the same brand) are used, the Rf values should be confirmed as per the validation process. The spotting technique also has significant effects on the TLC results. The spot size should be about 2 to 5 µL. It should be dried and placed above the mobile phase level.

### pH

The pH value of an injectable should be as close to the physiological pH as possible. The BP does not specify a method for testing the pH of the ^18^F–FDG. Some laboratories would use pH papers while others would use pH meters. It should be noted that the pH paper used should be verified with standard pH buffers, display a colour change for each 0.5 pH unit, and the pH value measured using pH paper is only an approximate [24]

### Chemical purity

The BP specifies the chemical purity FDG and 2–chloro–deoxyglucose (for acid hydrolysis synthesis only) to be determined by HPLC with a strong basic anion exchange column. The author has used a Carbopac™ column with good results, however, other commercially available strong basic anion exchange columns can perform equally well. The mobile phase is 0.1M NaOH and the flow rate is 1ml/min. Since NaOH absorbs carbon dioxide from air readily, it should be protected from air, stored in plastic containers and freshly prepared if possible. The Carbopac™ column is also very sensitive to carbonate ions. This adds to the importance of protecting the NaOH from air.

The test protocol includes injecting and run the HPLC of a reference standard solution and then run the HPLC of the test solution. The acceptance criteria is the area under the FDG peak of the test solution should be less than that of the reference solution. In theory, the reference material used should be of pharmacopeia grade. The USP has listed three USP grade FDG reference standards, but so far it has not been available commercially. One can only obtain non pharmacopeia grade FDG or 2–chloro–deoxyglucose from commercial vendors for preparation of reference solutions.

It is interesting to note that the BP states the preparation of glucose reference solution in addition to FDG and 2–chloro–deoxyglucose but does not require the reporting of glucose quantity presence in an ^18^F–FDG product.

The test of Kryptofix involves spotting the test solution and the reference standard on a TLC–SG plate and then develop the plate in a mixture of methanol and ammonia (9:1 v/v). The developed plate is then exposed to iodine vapor. The test solution spot should have a colour lighter than the reference solution spot. However, this TLC method is unreliable. The spots can be indistinct [24]. Alternatively, Kryptofix can be determined by placing the TLC plate in an iodine chamber directly or by GC [25].

### Residual Solvent

The BP lists only the determination of residual acetonitrile in the ^18^F–FDG product. But the BP does not specify the test method, although the description implies that a GC should be used.. The GC column should be used for aqueous solvent and the oven temperature should be constant. A flame ionisation detector is adequate. The actual temperature, carrier gas flow rate, and run time vary among different laboratories.

The BP does not mention any test of residual absolute ethanol. Since absolute ethanol is widely used in deferent ^18^F–FDG synthesis modules and GC test takes only a few minutes, it is better to measure the residual absolute ethanol concentration in the ^18^F–FDG product. Many laboratories adopt the USP limits of 0.05% or 5mg/mL

### Radionuclidic purity

The BP lists recording gamma spectrum and measuring half–life as two methods to determine the radionuclidic purity of a ^18^F–FDG product. Measurement of half–life can only confirm the presence of ^18^F. It does not reveal the percentage purity of the ^18^F^–^ present. The more accurate method is to obtain a gamma spectrum with a multi–channel analyzer after confirmation of ^18^F^–^ by measuring its half–life The BP allows the ^18^F–FDG to be released before the completion of this test.

Some experts doubt the necessity of carrying out a radionuclidic purity determination since its outcome is not crucial to patient welfare and imagine quality[24]. In fact, many laboratories measure the half–life of their ^18^F–FDG, but they do not obtain gamma spectra of their ^18^F–FDG products routinely.

### Radiochemical purity

The BP lists both HPLC method and TLC method for the determination of radiochemical purity. The method has been described in radiochemical identity under section *“(2) Identity (radionuclidic and radiochemical)”*

### Sterility

Sterility is to be tested by incubating the test sample with both Soybean Casein Digest Medium(SCDM) and Fluid Thioglycollate (FTM) Medium for 14 days at 37°C. Soybean Casein Digest Medium is a culture media for aerobic bacteria and fungi while FTM is a media for anaerobic bacteria. Growth Promotion Tests should be performed simultaneously. This test is performed by incubating “reference bacteria” in SCDM and FTM. Bacterial growth should be visible within a specified period of incubation (Table 3). Results of the growth promotion would indicate that the SCDM and FTM are capable of supporting bacterial growth, hence results of the sterility test are reliable. However, the US FDA has recommended a 30–hr window for ^18^F–FDG within which the sterility test must be started.

**Table 3:** Test microorganisms listed in BP suitable for Growth Promotion test

**Microorganism**		**Incubation**	
**Species**	**Strain**	**Incubation temperature**	**Maximum duration within which bacterial growth is visible**
Aerobic bacteria:			
Staphylcoccus aureus	ATCC 6538 CIP4.83 NCTC 10788 NCIMB 9518	30 to 35°C in FTM	3 days
Bacillus subtilis	ATCC6633 CIP52.62 NCIMB 8054		
Pseudomonas aeruginosa	ATCC 9027 NCIMB 8626 CIP82.118		
Anaerobic bacteria			
Clostridium sporogenes	ATCC 19404 CIP79.3	30 to 35°C in SCDM	3 days
Fungi			
Candida albicans	ATCC 10231 IP48.72 ATCC 2091 IP1180.79	30 to 35°C in FTM	5 days
Aspergillus niger	ATCC 16404		

Most PET facility would forward their samples to other microbiology laboratories for sterility test. A period of decay is necessary to ensure that the radioactivity level is not excessive. In many cases, a 24–hr window may not be long enough. Individual laboratories should establish their own protocols in this matter.

### Bacterial endotoxins (LAL test)

The bacterial endotoxins level is commonly tested using the gel–clot technique. The technique uses a lysate of amoebocytes from horseshoe crab, *Limulus polyphemus*. The addition of bacterial endotoxins to a lysate solution produces turbidity, precipitation or gelation of the mixture. Most commercially available endotoxin testing kits require an incubation period of 20 to 60 min. Hence, it is unlikely that the test can be completed before release of the product. The BP allows the release of the ^18^F–FDG before completion of the bacterial endotoxins test. Some PET facility would forward their samples to other microbiology laboratories for endotoxins test. As described earlier, a period of decay is necessary to ensure that the radioactivity level is not excessive.

Bacterial endotoxins level can also be determined by spectrophotmetry. The chromogenic method makes use of the colour change of a substrate produced by the formation of an enzyme which in turn results from the addition of endotxins to *Limulus polyphemus* lysate. Gram–negative bacterial endotoxins have been found to activate a proenzyme in *Limulus polyphemus* lysate. The rate of this activation reaction depends on the concentration of the endotoxins present. The activated proenzyme then catalyses the spitting of substrates added. The splitting of the substrates results in a colour change which can be monitored by spectrophotometry. Then time required for the appearance of the colour change is inversely proportional to the endotoxins concentration present. Hence, the endotoxins concentration can be determined by comparing the reaction time of a sample to a standard curve generated from a series of standards containing known concentrations of endotoxins [26, 27].

The endotoxins concentration in a sample can also be determined by measuring the turbidity change during the gel–clot formation using spectrophotometry. The time for onset of turbity is inversely related to the endotoxind concentration present. Endotoxind level in unknown sample can be determined by comparing the time required for turbidity onset to a standard curve generated from a series of standards with known endotoxins concentrations [28]. However, such method is extremely sensitive to interference from polysaccharide such as β–Glucans. Improved methods have been developed to reduce such interference [29].

### Filter membrane integrity test

This test is not required by BP and USP, but is required in the CMC section of US FDA [23]. Many laboratories have also included this test as one of their routine quality control tests of ^18^F–FDG.

Since the ^18^F–FDG is released and injected into patients before the sterility results are available, there is virtually no assurance of the product sterility. Filter membrane integrity test provide an indirect evident that the product is sterile. The argument is that if the integrity of the filter membrane is not compromised, the filter would have performed its function of removing any bacteria present in the ^18^F–FDG product.

**Figure 8:**
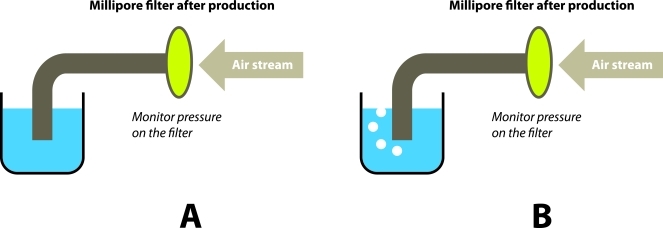
(A): Filter membrane is intact, no air passes through the membrane, no air bubble in water. (B): Filter membrane is broken or at bubble point, air passes through the membrane, air bubble in water. The bubble point should be higher than or equal to maximum pressure listed in the specification of the filter.

A few filter membrane integrity testing devices are available commercially. Some of them rather complicated and some of them are simple hand–held types. The mechanisms behind them are similar. A stream of air is passed through the devices to the filter, then to a reservoir of water. An indicator will show the pressure exerted on the filter membrane by the air stream. The filter membrane should be able to stand the maximum pressure indicated in the specification of the filter. If the membrane is broken the air stream will pass through the membrane into the water. Air bubble will then be seen. (Figure 8).

The biggest disadvantage of performing filter membrane integrity test is that usually the filter membranes are highly radioactive immediately after production of ^18^F–FDG. But allowing a 24–hr decay would defeat the purpose of providing an evident of sterility before injecting the ^18^F–FDG. Individual laboratories would have to develop their own protocols in this matter.

## CONCLUSION

Much of the current success in clinical PET can be attributed to the development of ^18^F–FDG. Synthesis of ^18^F–FDG is probably the most repeatable and highest yield in all PET radiopharmaceuticals synthesis. However, the future of PET would depend on the upcoming of new radiopharmaceuticals and the regulatory framework for the usage and approval of new PET drug products (e.g. NDA, IND etc). Synthesis, quality control and regulation of ^18^F–FDG become a model in the development new PET radiopharmaceuticals. Nucleophilic and electrophilic fluorinations are very common reactions to label compounds with ^18^F. The concept of using automatic synthesis modules is now a platform in PET radiopharmaceuticals synthesis. It is hope that this article will provide a brief review of ^18^F–FDG synthesis and quality control for those who are interested in development of PET radiopharmaceuticals. However, this article is only a concise review and not complete. Interested readers are encouraged to seek more detailed information
